# Replication Stress: A Review of Novel Targets to Enhance Radiosensitivity-From Bench to Clinic

**DOI:** 10.3389/fonc.2022.838637

**Published:** 2022-07-08

**Authors:** Yuewen Zhang, Lei Wu, Zhao Wang, Jinpeng Wang, Shrabasti Roychoudhury, Bartlomiej Tomasik, Gang Wu, Geng Wang, Xinrui Rao, Rui Zhou

**Affiliations:** ^1^ Cancer Center, Union Hospital, Tongji Medical College, Huazhong University of Science and Technology, Wuhan, China; ^2^ Division of Radiation and Genome Stability, Department of Radiation Oncology, Dana-Farber Cancer Institute, Harvard Medical School, Boston, MA, United States; ^3^ Department of Oncology and Radiotherapy, Medical University of Gdansk, Gdansk, Poland; ^4^ Institute of Radiation Oncology, Union Hospital, Tongji Medical College, Huazhong University of Science and Technology, Wuhan, China; ^5^ Department of Gastrointestinal Surgery, Union Hospital, Tongji Medical College, Huazhong University of Science and Technology, Wuhan, China

**Keywords:** replication stress, DNA damage repair, radiation therapy, radioresistance, radiosensitizer

## Abstract

DNA replication is a process fundamental in all living organisms in which deregulation, known as replication stress, often leads to genomic instability, a hallmark of cancer. Most malignant tumors sustain persistent proliferation and tolerate replication stress *via* increasing reliance to the replication stress response. So whilst replication stress induces genomic instability and tumorigenesis, the replication stress response exhibits a unique cancer-specific vulnerability that can be targeted to induce catastrophic cell proliferation. Radiation therapy, most used in cancer treatment, induces a plethora of DNA lesions that affect DNA integrity and, in-turn, DNA replication. Owing to radiation dose limitations for specific organs and tumor tissue resistance, the therapeutic window is narrow. Thus, a means to eliminate or reduce tumor radioresistance is urgently needed. Current research trends have highlighted the potential of combining replication stress regulators with radiation therapy to capitalize on the high replication stress of tumors. Here, we review the current body of evidence regarding the role of replication stress in tumor progression and discuss potential means of enhancing tumor radiosensitivity by targeting the replication stress response. We offer new insights into the possibility of combining radiation therapy with replication stress drugs for clinical use.

## Background

Although radiation therapy (RT) is used to treat ~50% of malignant tumors (1), it accounts for only 5% of the total cost of cancer patient care, making it the most cost-effective cancer treatment (2). RT is also an effective treatment for patients exhibiting a poor performance status who cannot tolerate surgery (3). Although new technologies, such as CyberKnife^®^, Tomotherapy^®^, and proton and heavy ion radiotherapy have been developed, radioresistance remains a crucial factor limiting our ability to cure cancer (4). Primary radioresistance can be caused by genomic or epigenetic changes in tumor cells, and radiation-induced genomic changes lead to secondary radioresistance, which is the most common cause of treatment failure and disease recurrence (5). Owing to limitations associated with normal tissue tolerance, increasing radiosensitivity in only cancer cells remains challenging.

Replication stress (RS) is the slowing or stalling of replication fork progression and is a major cause of genomic instability in cancer cells, which induces the accumulation of mutated and damaged DNA (6). In normal tissues, RS is a factor in the natural aging process (7). Cellular response to RS activates checkpoints to arrest cell cycle and repair DNA damage. Importantly, RS is selectively higher in cancer cells than in normal cells, and makes cancer cells more dependent on RS response pathways to survive (8, 9). Oncogene activation drives continuous proliferation, which is the basis for the generation of RS known as oncogene-induced RS. It is an important source of genome instability and might therefore be the basis of intratumor heterogeneity (10). Moreover, RS-induced DNA damage in tumors activates specific DNA damage repair pathways due to different genomic background cancer types. It also causes cells to enter mitosis with under-replicated regions that can cause genomic instability, thus potentially enhancing malignant behaviors (11). If the cellular response to RS is ineffective, then cells enter mitosis with an excess of damaged DNA, resulting in genomic instability or cell death due to mitotic catastrophe (12). These differences between normal and tumor cells suggest that targeting RS may contribute to the specific elimination of tumors (13).

RS has been highlighted as a hallmark of malignant tumor radiosensitivity (6, 14). Impaired responses to RS sensitize tumors to radiation (15), highlighting the importance of RS-aimed therapy for radiation treatment. Here, we summarize the current body of evidence concerning RS in cancer radiosensitivity, including known inhibitors and other potential targets. Treatments targeting RS-related pathways are suggested as an ideal radiosensitizer for cancer treatment.

## RS

Accurate DNA information is crucial for ensuring genomic stability. Conserving DNA integrity during DNA replication requires coordination between multiple cis- and trans-acting factors, such as regulating fork movement, nucleotide supply, transcription machinery, cellular checkpoints, and DNA repair pathways (16, 17). Here, we briefly summarize how RS occurs in malignant cells and the differences between cancer and normal cells, and then reason why RS is an ideal target for cancer treatment.

### Sources

**Figure 1:**
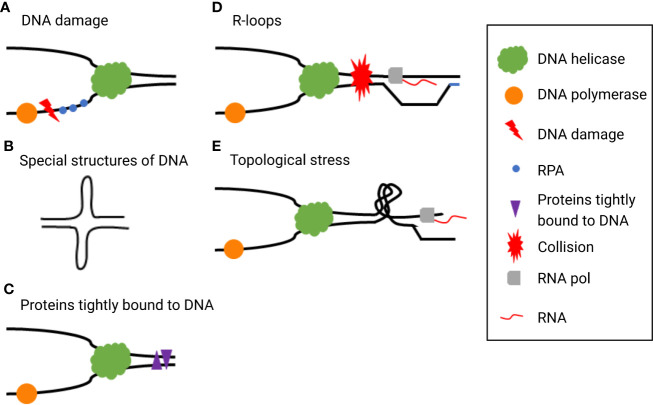
Typical exogenous and endogenous sources cause replication stress (RS), such as **(A)** DNA damage, **(B)** special DNA structures, **(C)** proteins tightly bound to DNA, **(D)** R-loops, and **(E)** topological stress.

Several major exogenous and endogenous factors that cause RS are listed here. Endogenous factors include alternative structures of DNA, centromeres, telomeres, DNA binding non-histones, replication, and transcription conflicts. All replication stressors affect the replication fork timing, causing the replication fork to slow down or even stall. Exogenous factors including DNA damage caused by radiation or cytotoxic substances, nucleotide loss, and abnormal replication, which activate DNA damage response (DDR) (**Figure 1**) (18).

### RS Responses

**Figure 2:**
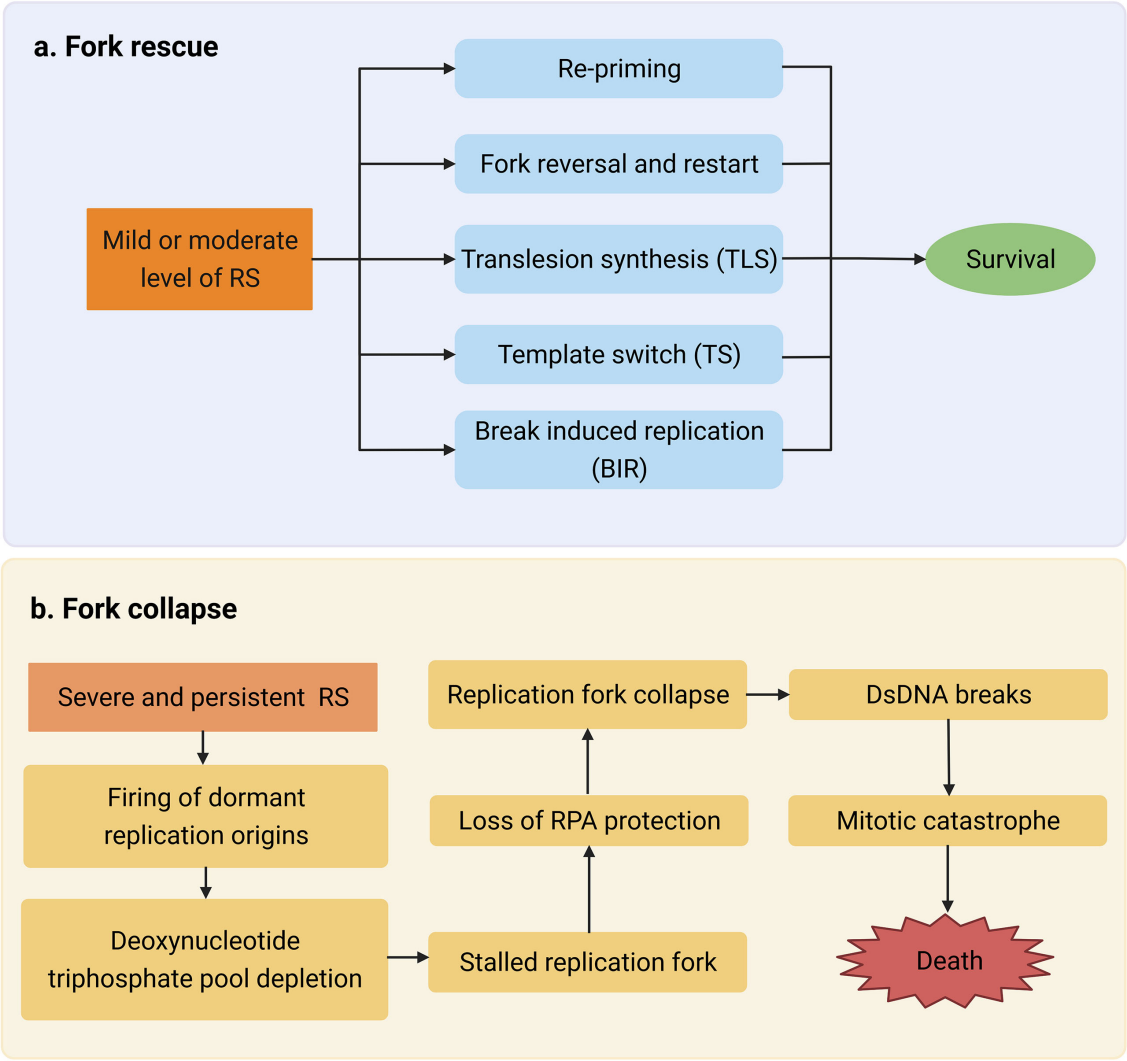
**(A)** Mild or moderate level of replication stress (RS) activates multiple mechanisms such as re-priming to repair DNA damage. **(B)** Severe and persistent RS leads to double-stranded DNA (dsDNA) break accumulation and eventually causes mitotic catastrophe which triggers cell death.

Cells have several strategies for dealing with RS called “RS responses”, including re-priming, fork reversal and restart, translation synthesis, template switching, and break-induced replication (16). RS response dysregulation is a typical characteristic of tumors, which may be caused by the loss of tumor suppressor factor or abnormal oncogene expression. Chronic RS increases the chance of breakage or gap formation in fragile sites, resulting in genomic instability, promoting further activation of oncogenes, and inducing malignant tumors in the early stage (8). Although mild or moderate levels of RS may induce tumorigenesis and promote tumor progression by accumulation genomic instability, in the event of severe and persistent RS, cells will finally develop mitotic disaster, senescence, or apoptosis (19). In the absence of active ataxia telangiectasia and rad3-related (ATR) and checkpoint kinase 1 (CHK1), replication forks cannot be stalled and thus continue to trigger dormant replication origins, leading to deoxynucleotide triphosphate pool depletion as well as slowing and stalling replication fork progression (12). When single-stranded DNA (ssDNA) is no longer protected by replication protein A (RPA), the replication fork collapses, resulting in double-strand breaks (DSBs). When these cells enter mitosis, unduplicated chromosomes trigger cell death through mitotic disasters (20, 21). Moreover, mutations produced during cancer development enhance RS and cause tumor cells to be hyper-dependent on RS response (18), which may be a potential target for cancer therapy (**Figure 2**).

## RS and Radioresistance in Cancer

**Figure 3:**
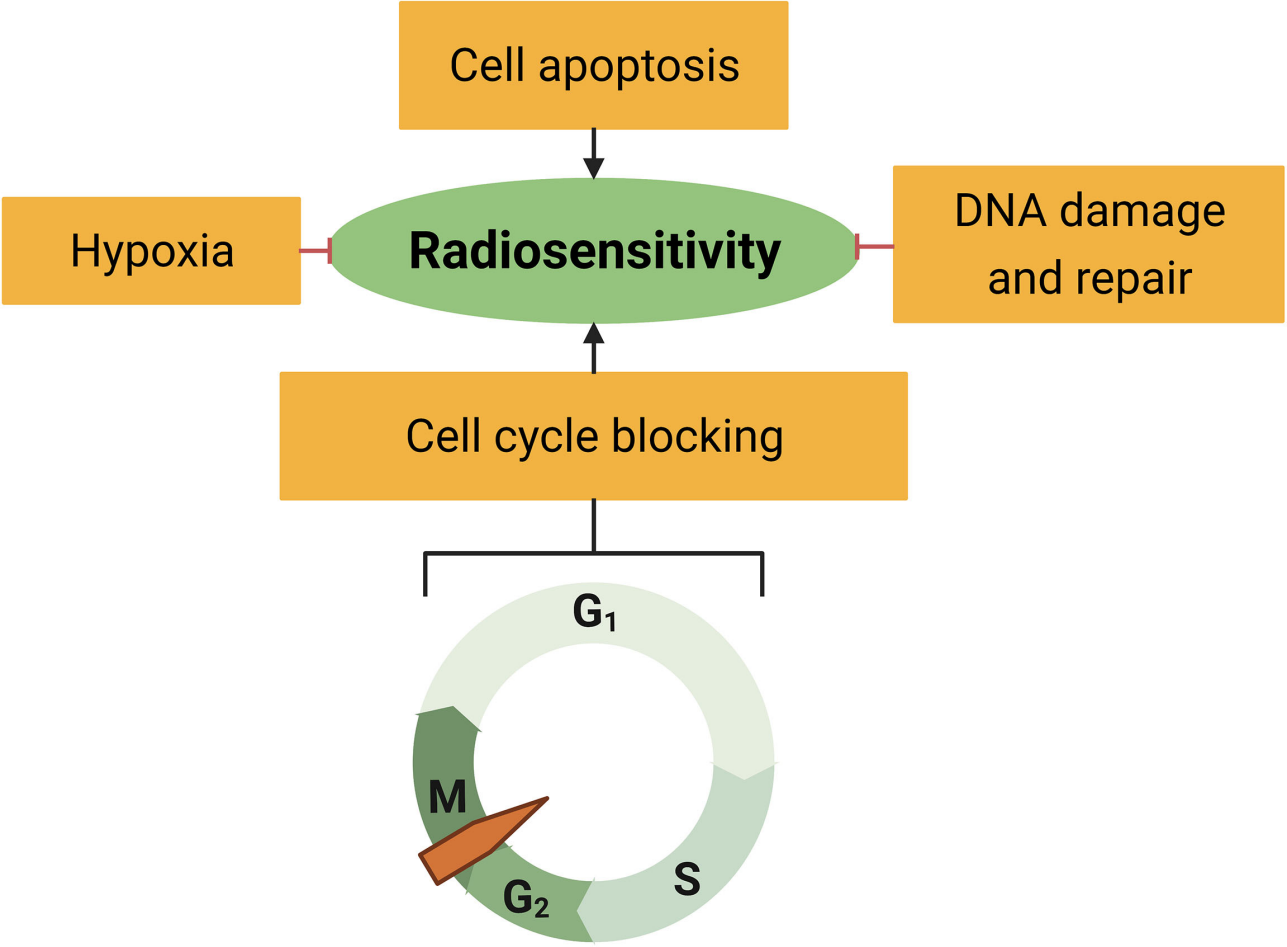
Radiosensitivity is associated with hypoxia, cell apoptosis, cell cycle distribution, and DNA damage response.

It is well-established that tumor radiation sensitivity greatly varies among individuals. As a result, some drugs have been reported to target multiple sensitivity or resistance factors (18, 22–24). Tumor radiosensitivity is mainly related to the intrinsic sensitivity of tumor cells and the cancer microenvironment (25). Here, we summarize the well-known mechanisms of radiation resistance and analyze the relationship between RS and the resistance factors (**Figure 3**).

### Hypoxia

Hypoxia is a common feature of malignant tumors resulting from rapid cell proliferation coupled with abnormal vasculature formation (26) and plays a pivotal role in tumor progression and treatment resistance (27). Hypoxia inducible factor (HIF), especially HIF-1, is the key regulator response to hypoxia. Clinical data have shown that eliminating the hypoxic state of tumors is an effective radiosensitizer (28, 29). Preclinical research has shown that NVX-108 increases tumor oxygen levels by 400%, significantly enhancing radio sensitivity (30). Phase I/II clinical trials have indicated the safety of NVX-108, and studies evaluating its efficacy are ongoing (29). Hypoxia also alters cell cycle response to ensure survival and minimal errors throughout cell division (31). Recent research claimed that hypoxia-induced RS was linked to the unfolded protein response (UPR) (32). There are few proteins that link hypoxic DDR and UPR, which suggests that they could be novel therapeutic targets to improve radiotherapy response (33, 34).

### Cell Apoptosis

Apoptosis is a key part of the intrinsic tumor suppression mechanism, which is triggered when proliferation becomes aberrant (35, 36). Targeting tumor cell apoptosis also contributes to radiosensitization. A high proportion of cells die through apoptosis, which is a positive indicator of radiosensitivity (37), and enhancing apoptosis effectively enhances tumor radiosensitivity. Knocking down remodeling and spacing factor-1 (RSF-1) enhanced the radiosensitivity of cervical cancer cells by redistributing the cell cycle, inducing cell apoptosis, and eventually inhibiting cell proliferation (38). Astaxanthin enhances irradiation-induced apoptosis in esophageal squamous cell carcinoma cells (39). Deficient RS response also leads to cell apoptosis, which suggests a role as a synergistic factor to RT (40).

### Cell Cycle Distribution

The cell cycle distribution of cancer cells affects radio sensitization, especially for some cancer types that depend more on other DDR pathways rather than homologous repair (HR) (41). In different cell cycles, the differences in chromosome structure lead to unequal radiosensitivity. Clinicians believe G2/M is the most sensitive phase since the radiation induces more complex damage that induce longer cell cycle arrest and therefore need proficient HR for repair (42). Meanwhile, the damage that occurs during G2/M can more easily cause premature entry into mitosis, which can lead to a higher possibility of passing incorrect genomic information to the next generation, or even cause mitotic catastrophe directly (43). Eurycomalactone, an active quassinoid isolated from *Eurycoma longifolia*, has been shown to sensitize non-small cell lung cancer cells to X-rays through a G2/M block (44). Further studies have focused on the G2/M arrest after receiving radiation. When DDR is activated, it temporarily stops the cell cycle to provide more time for repair, or if the damage is too severe, induces apoptosis. Eliminating the radiation-induced G2/M arrest or forcing damage cells to enter into mitosis both sensitizes cancer cells to radiation treatment (45, 46). This cell-cycle-dependent radiosensitization mechanism provides potential directions for further research into radiosensitizers.

### DNA Damage and Repair

Cells respond to DNA damage by activating the DDR pathway. Abnormal activation of DDR in tumor cells leads to the generation of radiotherapy resistance (46). High RS also leads to DNA damage and activate the DDR pathway. The five major DNA repair pathways are base excision repair, nucleotide excision repair, mismatch repair, HR, and non-homologous end joining (NHEJ). Any impaired pathway can be compensated for by the overactivation of other pathways (47). These compensatory mechanisms in tumor cells lead to different responses to treatment with DNA damage agents, as well as RT (1, 48). Both RS and radiation activate a similar DDR pathway, providing the possibility of a synergistic effect of targeting RS with RT (49, 50).

## Targeting RS as Radiation Sensitizer

**Figure 4:**
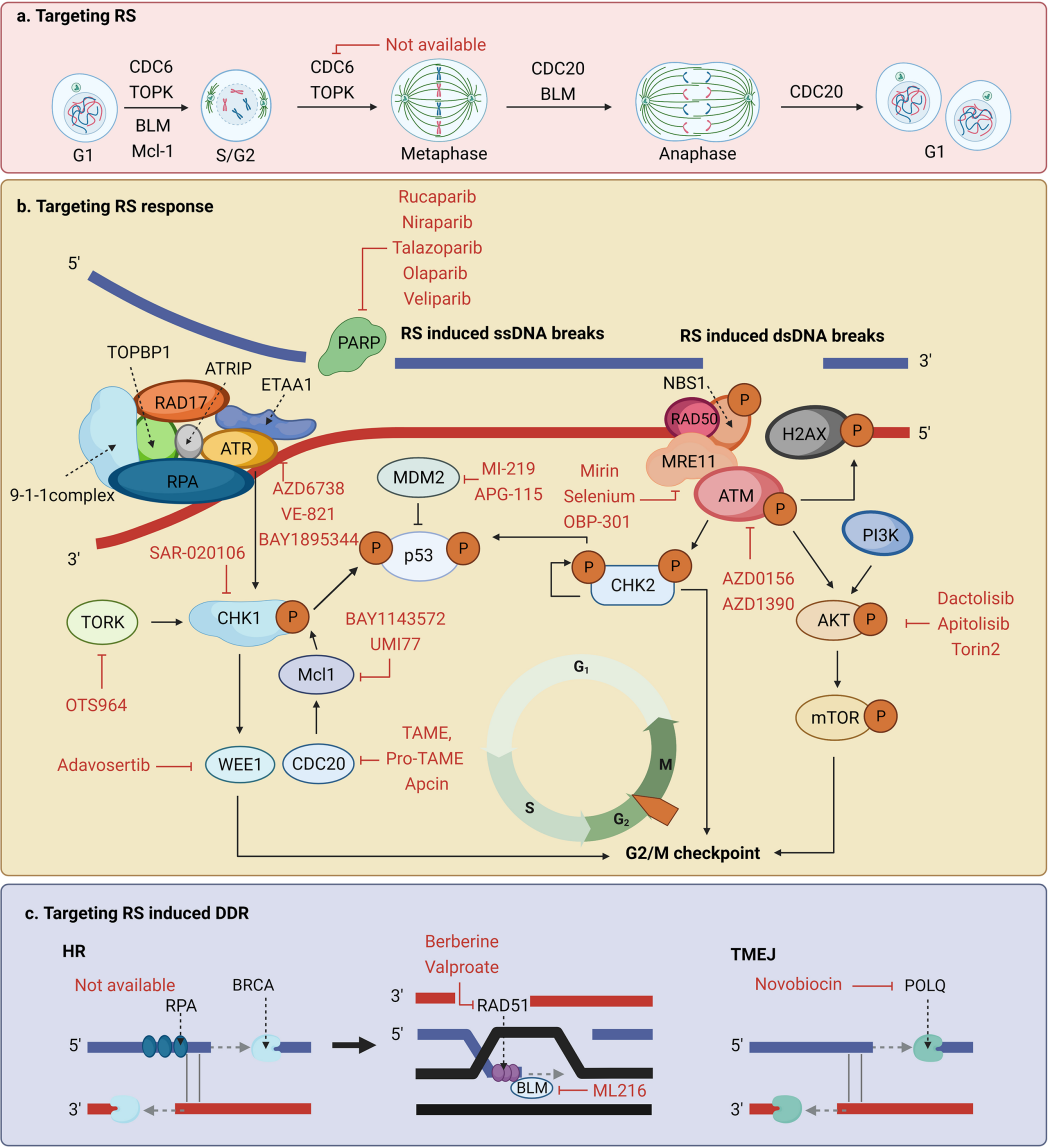
Potential targets and corresponding inhibitors of **(A)** the replication stress (RS), **(B)** the RS response, or **(C)** RS-induced DNA damage response (DDR) that have been previously reported.

Cancer cells relying more on RS response than normal cells to survive provides a potential target of anti-tumor treatment sensitization (51). In this section, we summarized and discussed the specific application of reagents targeting the RS response or RS-induced DDR that have already been demonstrated to be effective or have the potential to enhance tumor radiosensitization (**Table 1**, **Figure 4**).

**Table 1:** Targeting replication stress as radiation sensitizer.

Targeted Marker	Mechanism	Drug	Phase	Details (Including NCT Number)	Status
**Inducing exorbitant RS**					
CDC6	Decreased CDC6 expression in tumor cells effectively inhibits tumor cell growth and promotes apoptosis by preventing G1/S and S/G2 transition.	–	–	–	–
TOPK	TOPK sensitizes cancer cells to radiotherapy, owing to the preservation of irradiation-induced damage and reduced tolerance to RS.	–	–	–	–
CDC20	Reduced CDC20 expression disrupts the APC-CDC20 interaction and shows great effect on suppressing tumor proliferating and metastasis.	TAME	–	–	–
		pro-TAME	–	–	–
		Apcin	–	–	–
Mcl-1	Mcl-1 blocks radiation-induced apoptosis and inhibits clonogenic cell death.	BAY1143572 (Atuveciclib)	Phase I	Phase I Dose Escalation of BAY1143572 in Subjects With Acute Leukemia (NCT02345382)	Completed
			Phase I	Open Label Phase I Dose Escalation Study With BAY1143572 in Patients With Advanced Cancer (NCT01938638)	Completed
		UMI77	–	–	–
**Targeting RS response**					
PARP	Inhibition of PARP forces PARP to trap onto DNA thus preventing replication restart, causing RS-induced DNA damage.	Rucaparib (AG014699)	Phase I	A Study of Rucaparib Administered With Radiation in Patients With Triple Negative Breast Cancer With an Incomplete Response Following Chemotherapy (NCT03542175)	Recruiting
		Niraparib (MK-4827, Zejula)	Phase I/II	A Safety Study Adding Niraparib and Dostarlimab to Radiation Therapy for Rectal Cancers (NCT04926324)	Not yet recruiting
			Phase II	The Efficacy and Safety of Radiotherapy Plus Niraparib and Toripalimab in Patients With Recurrent Small Cell Lung Cancer (NCT05162196)	Not yet recruiting
			Phase I/II	Study of Niraparib With Radiotherapy for Treatment of Metastatic Invasive Carcinoma of the Cervix (NCT03644342)	Recruiting
			Phase II	Radiation, Immunotherapy and PARP Inhibitor in Triple Negative Breast Cancer (NCT04837209)	Recruiting
			Phase II	Niraparib With Standard Combination Radiation Therapy and Androgen Deprivation Therapy in Treating Patients With High Risk Prostate Cancer (NCT04037254)	Recruiting
			Phase II	Androgen Ablation Therapy With or Without Niraparib After Radiation Therapy for the Treatment of High-Risk Localized or Locally Advanced Prostate Cancer (NCT04947254)	Recruiting
			Phase II	Niraparib Combined With Radiotherapy in rGBM (NCT04715620)	Recruiting
			Phase II	Niraparib + Dostarlimab + RT in Pancreatic Cancer (NCT04409002)	Active, not recruiting
			Phase I/II	A Multi-Center Trial of Androgen Suppression With Abiraterone Acetate, Leuprolide, PARP Inhibition and Stereotactic Body Radiotherapy in Prostate Cancer (NCT04194554)	Recruiting
		Talazoparib (BMN673, Talzenna)	Phase I	Talazoparib and Radiation Therapy in Treating Patients With Locally Recurrent Gynecologic Cancers (NCT03968406)	Recruiting
			Phase II	A Study to Evaluate TAlazoparib, Radiotherapy and Atezolizumab in gBRCA 1/2 Negative Patients With PD-L1+ Metastatic Triple Negative Breast Cancer (NCT04690855)	Recruiting
			Phase I	Talazoparib and Thoracic RT for ES-SCLC (NCT04170946)	Recruiting
		Olaparib (AZD2281, KU0059436)	Phase I	Olaparib & Radiation Therapy for Patients Triple Negative Breast Cancer (TNBC) (NCT03109080)	Active, not recruiting
			Phase I/II	Phase I/IIa Study of Concomitant Radiotherapy With Olaparib and Temozolomide in Unresectable High Grade Gliomas Patients (NCT03212742)	Recruiting
			Phase II	Focal Radiation With Pulsed Systemic Therapy of Abiraterone, Androgen Deprivation Therapy (ADT), Lynparza Towards Castration Sensitive Oligometastatic Prostate Cancer (FAALCON) (NCT04748042)	Recruiting
			Phase II	Radiation Therapy With or Without Olaparib in Treating Patients With Inflammatory Breast Cancer (NCT03598257)	Recruiting
			Phase I	Study of Olaparib With Radiation Therapy and Cetuximab in Advanced Head and Neck Cancer With Heavy Smoking History (NCT01758731)	Completed
			Phase I	Olaparib and Radiotherapy in Inoperable Breast Cancer (NCT02227082)	Completed
			Phase I	Olaparib and Radiotherapy in Head and Neck Cancer (NCT02229656)	Active, not recruiting
			Phase II	A Study of Radiation Therapy With Pembrolizumab and Olaparib in Women Who Have Triple-Negative Breast Cancer (NCT04683679)	Recruiting
			Phase I	A Study of Olaparib and Low Dose Radiotherapy for Small Cell Lung Cancer (NCT03532880)	Recruiting
			Phase I	Radiotherapy & Olaparib in COmbination for Carcinoma of the Oesophagus (NCT01460888)	Unknown
			Phase I	A Study of Olaparib With Concomitant Radiotherapy in Locally Advanced/Unresectable Soft-tissue Sarcoma (NCT02787642)	Recruiting
			Phase I/II	Olaparib and Durvalumab With Carboplatin, Etoposide, and/or Radiation Therapy for the Treatment of Extensive-Stage Small Cell Lung Cancer, PRIO Trial (NCT04728230)	Recruiting
			Phase I	Radiotherapy and Durvalumab/Durvalumab Combo (Tremelimumab/Olaparid) for Small Cell Lung Cancer (NCT03923270)	Recruiting
			Phase I	Olaparib Dose Escalating Trial + Concurrent RT With or Without Cisplatin in Locally Advanced NSCLC (NCT01562210)	Completed
			Phase I	A Study to Investigate Biomarker Effects of Pre-Surgical Treatment With DNA Damage Repair (DDR) Agents in Patients With Head and Neck Squamous Cell Carcinoma (HNSCC) (NCT03022409)	Completed
			Phase I	A Platform Study of Novel Agents in Combination With Radiotherapy in NSCLC (NCT04550104)	Recruiting
			Phase I/II	Lu-177-DOTATATE (Lutathera) in Combination With Olaparib in Inoperable Gastroenteropancreatico Neuroendocrine Tumors (GEP-NET) (NCT04086485)	Not yet recruiting
			Phase I	Phase I Study of Olaparib With Cisplatin Based Chemoradiotherapy in Squamous Cell Carcinoma of the Head and Neck (NCT01491139)	Withdrawn
			Phase II/III	Refining Adjuvant Treatment IN Endometrial Cancer Based On Molecular Features (NCT05255653)	Not yet recruiting
		Veliparib (ABT-888, NSC 737664)	Phase I	A Phase I Study of ABT-888 in Combination With Conventional Whole Brain Radiation Therapy (WBRT) in Cancer Patients With Brain Metastases (NCT00649207)	Completed
			Phase I	A Clinical Study Conducted in Multiple Centers Evaluating Escalating Doses of Veliparib in Combination With Capecitabine and Radiation in Patients With Locally Advanced Rectal Cancer (NCT01589419)	Completed
			Phase I	Veliparib in Combination With Gemcitabine and Intensity Modulated Radiation Therapy in Patients With Pancreatic Cancer (NCT01908478)	Completed
			Phase I/II	Veliparib, Radiation Therapy, and Temozolomide in Treating Younger Patients With Newly Diagnosed Diffuse Pontine Gliomas ( NCT01514201)	Completed
			Phase II	Comparison of Veliparib and Whole Brain Radiation Therapy (WBRT) Versus Placebo and WBRT in Adults With Brain Metastases From Non-Small Cell Lung Cancer	Completed
			Phase I	Veliparib and Radiation Therapy in Treating Patients With Advanced Solid Malignancies With Peritoneal Carcinomatosis, Epithelial Ovarian, Fallopian, or Primary Peritoneal Cancer (NCT01264432)	Completed
			Phase I	Veliparib With Radiation Therapy in Patients With Inflammatory or Loco-regionally Recurrent Breast Cancer (NCT01477489)	Completed
			Phase I	Pre-Operative Radiation and Veliparib for Breast Cancer (NCT01618357)	Recruiting
			Phase II	Veliparib, Radiation Therapy, and Temozolomide in Treating Patients With Newly Diagnosed Malignant Glioma Without H3 K27M or BRAFV600 Mutations (NCT03581292)	Active, not recruiting
			Phase I	ABT-888, Radiation Therapy, and Temozolomide in Treating Patients With Newly Diagnosed Glioblastoma Multiforme (NCT00770471)	Completed
			Phase I/II	Veliparib With or Without Radiation Therapy, Carboplatin, and Paclitaxel in Patients With Stage III Non-small Cell Lung Cancer That Cannot Be Removed by Surgery (NCT01386385)	Active, not recruiting
			Phase I/II	A Study Evaluating the Efficacy and Tolerability of Veliparib in Combination With Paclitaxel/Carboplatin-Based Chemoradiotherapy Followed by Veliparib and Paclitaxel/Carboplatin Consolidation in Adults With Stage III Non-Small Cell Lung Cancer (NSCLC) ( NCT02412371)	Terminated
RPA	Overexpression of RPA significantly increases the radiation resistance in multiple cancer types.	–	–	–	–
TopBP1	TopBP1 is known to form phase-separated nuclear condensates that amplify ATR activity to CHK1 and slow down replication forks.	–	–	–	–
ATR-CHK1	Inhibition of ATR-related signaling pathways increases cell apoptosis and effectively improves tumor radiosensitivity.	AZD6738 (Ceralasertib)	Phase I	Phase I Study to Assess Safety of AZD6738 Alone and in Combination With Radiotherapy in Patients With Solid Tumours (NCT02223923)	Unknown
			Phase I	A Study to Investigate Biomarker Effects of Pre-Surgical Treatment With DNA Damage Repair (DDR) Agents in Patients With Head and Neck Squamous Cell Carcinoma (HNSCC) (NCT03022409)	Completed
		VE-821	–	–	–
		SAR-020106	–	–	–
		BAY1895344 (Elimusertib)	Phase I	First-in-human Study of ATR Inhibitor BAY1895344 in Patients With Advanced Solid Tumors and Lymphomas (NCT03188965)	Active, not recruiting
			Phase I	Testing the Addition of an Anti-cancer Drug, BAY1895344, With Radiation Therapy to the Usual Pembrolizumab Treatment for Recurrent Head and Neck Cancer (NCT04576091)	Recruiting
RAD51	Inhibition of RAD51 induces RS to promote apoptosis.	Berberine	–	–	–
		Valproate	Phase II	Valproic Acid, Radiation, and Bevacizumab in Children With High Grade Gliomas or Diffuse Intrinsic Pontine Glioma (NCT00879437)	Completed
			Phase I/II	Preoperative Valproic Acid and Radiation Therapy for Rectal Cancer (NCT01898104)	Recruiting
			Phase II	Valproic Acid With Temozolomide and Radiation Therapy to Treat Brain Tumors (NCT00302159)	Completed
			Phase I	Phase I Study of Temozolomide, Valproic Acid and Radiation Therapy in Patients With Brain Metastases (NCT00437957)	Terminated
			Phase I/II	Valproic Acid With Chemoradiotherapy for Non-Small-Cell Lung Cancer (NCT01203735)	Unknown
BLM	The high expression of BLM is a poor prognostic biomarker for multiple cancers. Though there’s no data published about the links between BLM inhibitor and radiation sensitivity till now, it’s a promising target worth further research.	ML216 (CID-49852229)	–	–	–
WEE1	Inhibition of WEE1 impairs RS response activated by ATR, and thus increasing tumor cell radiosensitivity.	AZD1775 (Adavosertib, MK-1775)	Phase I	Adavosertib, Radiation Therapy, and Temozolomide in Treating Patients With Newly Diagnosed or Recurrent Glioblastoma (NCT01849146)	Active, not recruiting
			Phase I	Testing the Addition of an Anti-cancer Drug, Adavosertib, to Radiation Therapy for Patients With Incurable Esophageal and Gastroesophageal Junction Cancers (NCT04460937)	Suspended
			Phase I	Adavosertib and Local Radiation Therapy in Treating Children With Newly Diagnosed Diffuse Intrinsic Pontine Gliomas (NCT01922076)	Active, not recruiting
			Phase I	Testing AZD1775 inC Combination With Radiotherapy and Chemotherapy in Cervical, Upper Vaginal and Uterine Cancers (NCT03345784)	Active, not recruiting
			Phase I	Dose-escalating AZD1775 + Concurrent Radiation + Cisplatin for Intermediate/High Risk HNSCC (NCT02585973)	Completed
			Phase I/II	Dose Escalation Trial of AZD1775 and Gemcitabine (+Radiation) for Unresectable Adenocarcinoma of the Pancreas (NCT02037230)	Completed
			Phase I	WEE1 Inhibitor With Cisplatin and Radiotherapy: A Trial in Head and Neck Cancer (NCT03028766)	Completed
**Targeting RS induced DDR**					
p53	Activation of p53 activates cell cycle block and apoptosis.	–	–	–	–
MRE11	Low MRE11 expression reduces phosphorylated DNA-PKcs expression, further increases tumor radiosensitivity.	Mirin	–	–	–
		Selenium	Phase II	Capecitabine, Oxaliplatin, Selenomethionine, and Radiation Therapy in Treating Patients Undergoing Surgery For Newly Diagnosed Stage II or III Rectal Adenocarcinoma (NCT00625183)	Terminated
			Phase II	Carboplatin, Paclitaxel, Selenomethionine, and Radiation Therapy in Treating Patients With Stage III Non-Small Cell Lung Cancer That Cannot Be Removed by Surgery (NCT00526890)	Terminated
			Phase II	Selenomethionine in Reducing Mucositis in Patients With Locally Advanced Head and Neck Cancer Who Are Receiving Cisplatin and Radiation Therapy (NCT01682031)	Terminated
			Phase II	Selenomethionine and Finasteride Before Surgery or Radiation Therapy in Treating Patients With Stage I or Stage II Prostate Cancer (NCT00736645)	Completed
			Phase II	Selenomethionine in Treating Patients Undergoing Surgery or Internal Radiation Therapy for Stage I or Stage II Prostate Cancer (NCT00736164)	Withdrawn
		OBP-301 (Telomelysin)	Phase I	A Study of OBP-301 With Radiation Therapy in Patients With Esophageal Cancer (NCT03213054)	Unknown
ATM-CHK2	Deficiency of ATM shows radiation sensitizer effect in multiple cancer types. The effect of ATM on radiation sensitivity is more depend on cell cycle regulation rather than DDR pathway.	AZD0156	–	–	–
		AZD1390	Phase I	A Study to Assess the Safety and Tolerability of AZD1390 Given With Radiation Therapy in Patients With Brain Cancer (NCT03423628)	Recruiting
			Early Phase 1	AZD1390 in Recurrent Grade IV Glioma Patients (NCT05182905)	Recruiting
			Phase I	A Platform Study of Novel Agents in Combination With Radiotherapy in NSCLC (NCT04550104)	Recruiting
			Phase I	Sarcomas and DDR-Inhibition; a Combined Modality Study (NCT05116254)	Not yet recruiting
MDM2	Inhibition of MDM2 phosphorylation leads to cell apoptosis and cell cycle arrest, thus repressing tumor cell proliferation.	MI-219	–	–	–
		APG-115 (Alrizomadlin)	–	–	–
POLQ	Reduced POLQ expression inhibits DSB repair and tumor cell survival.	Novobiocin	–	–	–
BRCA	Mutations in BRCA is synthetic lethal with PARP inhibition.	–	–	–	–
PI3K/AKT/mTOR	Inhibition of PI3K/AKT/mTOR signaling pathway leads to cell cycle arrest in the G2/M phase and reduces tumor cell radio-resistance.	Dactolisib (BEZ235, NVP-BEZ235)	–	–	–
		Apitolisib (GDC-0980, RG7422, GNE 390)	–	–	–
		Torin2	–	–	–
**Others**					
Ubiquitin and SUMO	SUMO/ubiquitin equilibrium at active DNA replication forks controls CDK1 activation.	–	–	–	–
UPR	Activated UPR reduces the oxidative phosphorylation thus impairing cell cycle arrest and DNA repair factors after radiation also enhance radiation induced cell death.	ONC201 (TIC10)	Phase II	Combination Therapy for the Treatment of Diffuse Midline Gliomas (NCT05009992)	Recruiting
			Phase I	ONC201 and Radiation Therapy Before Surgery for the Treatment of Recurrent Glioblastoma (NCT04854044)	Withdrawn

Data retrieved from: https://clinicaltrials.gov/ct2/home Retrieval data 04/19/2022.

RS, replication stress; DDR, DNA damage response; CDC6, cell division cycle 6 homologue; TOPK, t-lymphoid-activated killer (T-LAK) cell-derived protein kinase; CDC20, cell division cycle protein 20 homologue; TAME, tosyl-L-arginine methyl ester; Mcl-1, myeloid cell leukemia sequence 1; PARP, poly (ADP-ribose) polymerases; RPA, replication protein A; TopBP1, topoisomerase II-binding protein 1; ATR, ataxia telangiectasia and rad3-related; CHK, checkpoint kinase; MRE11, meiotic recombination 11; ATM, ataxia telangiectasia mutated; MDM2, mouse double minute 2; POLQ, DNA polymerase theta; BRCA, breast cancer related protein; PI3K, phosphoinositide 3-kinase; AKT, protein kinase B; mTOR, mammalian target of rapamycin; SUMO, small ubiquitin-like modifier; UPR, unfolded protein response.

### Inducing Exorbitant RS

In this section, we summarized and discussed the known factors that contribute to the normal DNA replication process. Losing control of them triggers RS thus synthetically sensitizing radiation.

#### CDC6

Cell division cycle 6 homologue (CDC6) is an important regulator of DNA replication in eukaryotic cells (52, 53) involved in replication complex assembly during G1 phase. Replication fork stall accumulation caused by RS triggers G2/M checkpoint activation. CDC6 promotes the response of the G2/M checkpoint (54) and is positively correlated with tumor progression. Decreased CDC6 expression in tumor cells effectively inhibits tumor cell growth and promotes apoptosis by preventing G1/S and S/G2 transition (55). CDC6 overexpression has been observed in radiation-resistant cells, contributing to an increase in radiation resistance in cancer cells (56). CDC6 downregulation enhanced cisplatin-resistant bladder cancer cell sensitivity in a clinical trial, which is also related to DSB damage (57). Therefore, CDC6 inhibition in tumor cells might be an effective target for enhancing tumor radiosensitivity. Although CDC6 has druggable sites for a chemical molecular, it is an essential protein in most cell lines that makes it difficult for clinical transformation (58). Thus, further study on the regulatory mechanism of CDC6 in radiation resistance will help to develop clinical practical drugs in the future.

#### TOPK

T-lymphoid-activated killer (T-LAK) cell-derived protein kinase (TOPK) is a mitogen-activated protein kinase kinase-like kinase that plays an important role in cell cycle regulation. TOPK overexpression is a pathophysiological feature in different tumors (59).

TOPK knockdown does not change the radiation response of normal tissues but significantly enhances cancer cell radiosensitivity, and TOPK disruption may lead to tumor-specific radiosensitivity (60). Thus, TOPK, as a cancer-specific biomarker and biochemical target, may enhance the efficacy of cancer treatment while causing minimal damage to normal tissues (59). TOPK was found to enhance tumor radiosensitivity by enhancing intratumor RS (61). Further experiments demonstrated that TOPK helps to restart the stopped replication fork. However, when TOPK was depleted, increased levels of stalled replication forks were observed, with or without external DNA damage (61). Therefore, TOPK suppression increases internal replication damage. Owing to the preservation of irradiation-induced damage and reduced tolerance to RS, TOPK sensitizes cancer cells to radiotherapy.

TOPK interacts with CHK1 and cell division cycle 25 homologue C (CDC25C) complex (key participants in the replication of the damage induced) (61). It facilitates mitotic progression at the G2/M checkpoint *via* cyclin-dependent kinase 1 (CDK1), and also occurs in response to replication stressors (such as irradiation) by influencing the action of key intermediates such as CHK1 (61). Therefore, the synergistic effect of TOPK inhibition and radiotherapy is likely to produce DSBs after replication. However, unlike CHK1, the toxicity of TOPK inhibitors is limited in normal tissues due to low expression. Therefore, TOPK appears to be a promising target for further research.

#### CDC20

Cell division cycle 20 homologue (CDC20) has important functions in chromosome segregation and mitotic exit. It is the target of the spindle assembly checkpoint (SAC) and the key cofactor of the anaphase-promoting complex or cyclosome (APC/C) E3 ubiquitin ligase, thus regulating APC/C ubiquitin activity on specific substrates for their subsequent degradation by the proteasome (62). CDC20 is overexpressed in tumor cells and acts as a poor prognostic factor in multiple cancers (63, 64). It further increased after radiation and has been reported to increase radiation resistance *via* regulating B-cell lymphoma-2 (Bcl-2)/Bcl-2-associated X protein (Bax), forkhead box proteins O1 (FoxO1), or myeloid cell leukemia sequence 1 (Mcl-1)/p-CHK1 in different cancer types (65–67). Suppression of CDC20 expression reverses the radioresistance (65–67). There are multiple available inhibitors of CDC20, including tosyl-L-arginine methyl ester (TAME), Pro-TAME, and apcin. Their main effects involve disrupting the APC-CDC20 interaction (68, 69). Some of them showed great efficacy in suppress tumor proliferating and metastasis (70, 71). However, there has been no evidence on the effects of the CDC20 inhibitors on radiosensitivity. Therefore, CDC20 could be a potential target as a radiosensitizer, but more evidence in future studies is needed.

#### Mcl-1

As the first anti-apoptotic protein in the Bcl-2 family, Mcl-1 is regulated by the cell cycle and reach peak expression levels in the S/G2 phase. It acts as a functional switch in selecting between HR and NHEJ pathways after DNA damage (72). It blocks radiation-induced apoptosis and inhibits clonogenic cell death (73). Targeting Mcl-1 by a small molecule enhances RS sensitivity to cancer therapy (72). BAY1143572 downregulated Mcl-1 by inhibiting binding of HIF-1α to the Mcl-1 promoter (74). UMI77 is a selective inhibitor of Mcl-1 that dissociates Mcl-1 from the pro-apoptotic protein Bak and produced significant radiosensitization in pancreas cancers (75).

### Targeting RS Response

Here, we summarize important RS response factors that are essential for cells to survive. Inhibition of these factors leads to uncontrolled replication collapse and even mitotic catastrophe, which makes them ideal targets for radiosensitization.

#### PARP

Poly (ADP-ribose) polymerases (PARPs) are involved in DDR and recruit DNA repair proteins to damaged sites by catalyzing ADP-ribosylation, leading to the formation of poly (ADP-ribose) polymers (76). PARP1, the most abundant PARP, plays a similar role to PARP2 in the DDR process and is an important regulator of fork reversal (77). Inhibition of PARP directly increases the speed of fork elongation and does not cause fork stalling, which contrasts with the accepted model in which inhibitors of PARP induce fork stalling and collapse. Aberrant acceleration of fork progression by 40% above the normal velocity leads to DNA damage (78).

However, the effects of PARP inhibitor do not directly decrease the expression of PARP. Rather, the inhibitor forces PARP to become stuck on DNA, thus preventing replication restart and causing RS-induced DNA damage (79). It was also linked to decreased replication fork length with greater ssDNA gaps, which in turn cause more genomic instability at G2/M (80). With all the evidence of PARP inhibitors in RS-induced DNA damage, researchers have reported on various preclinical models of combination therapy with PARP inhibitors and ionizing radiation (IR) (81). Olaparib, a PARP inhibitor that has been widely used in cancer treatment, has been reported to have strong tumor-specific radiosensitization effects (82, 83).

#### RPA

The RPA complex is one of the first responders to coordinate DNA replication (18, 84). It consists of three subunits, RPA1 (RPA70), RPA2 (RPA32), and RPA3 (RPA14), which are essential to protect ssDNA at replication forks and recruits DNA polymerases α, δ, and ϵ for the initiation and elongation steps of DNA replication (84). It has been reported that RPA1 phosphorylation upon RS decreases the ubiquitination of chromatin-loaded RPA1, leading to an accumulation of RPA1 on stalled replication forks. This helps the DNA-binding domains of RPA2 to bind with RPA1-coated ssDNA, thus contributing to increased RPA2 binding stability (85). Loss of RPA accelerates fork breakage, whereas overexpression of RPA is sufficient to delay a “replication catastrophe” (86). It also plays an important role in DDR in relation to the HR pathway (87). Furthermore, overexpression of RPA significantly increases the radiation resistance in multiple cancer types (88–90). However, there has been no reported inhibitors of RPA because it is an essential protein to all cells. Furthermore, it is a downstream factor of ATR, and thus the regulation of ATR may produce similar effects (86). RING finger and WD repeat domain 3 (RFWD3)-mediated ubiquitination of RPA helps to remove RPA from the damage site, which is a crucial step for HR (91), and thus provides a possible target for increasing radiation sensitivity *via* ubiquitination regulation.

#### TopBP1

DNA topoisomerase II-binding protein 1 (TopBP1) serves as a scaffold to assemble protein complexes in a phosphorylation-dependent manner *via* its multiple breast cancer C-terminal (BRCT) repeats. It is repurposed to scaffold different processes dependent on cell cycle-regulated changes in phosphorylation of target proteins (92). It is known to form phase-separated nuclear condensates that amplifies ATR activity to CHK1 and slow down replication forks (93). TopBP1 also stabilized bloom syndrome helicase (BLM) to maintain genome stability (94). It is often overexpressed in cancer and can bypass control by CDK2 to interact with treslin, leading to enhanced DNA replication (95).

However, it has been reported that at low levels, TopBP1 activates ATR/CHK1, but once TopBP1 protein accumulates above an optimal level, it paradoxically leads to lower activation of ATR/CHK1. This is due to the perturbation of ATR-TopBP1 interaction and ATR chromatin loading by excessive TopBP1. Depletion of TopBP1 in some specific cancer cells enhanced ATR/CHK1 activation and S-phase checkpoint response after RS (96). Thus, simply inhibiting TopBP1 may lead to unexpected results, which makes it not an ideal target for radiation sensitization.

#### ATR-CHK1

ATR of the phosphoinositide 3-kinase (PI3K) family is a central regulator of RS. After ssDNA fragments are coated with PRA, ATR and ATR-interacting protein are recruited and activated. It further phosphorylates various proteins, including CHK1 kinase, which inhibits mitotic entry and dormant origin activation. Mitotic entry is inhibited by CDC25 phosphatase phosphorylation, which prevents subsequent mitotic CDK activation (97). Cancer genome sequencing showed a very low ATR or CHK1 mutation or deletion frequency. Instead, these genes are often amplified in cancer cells, probably because they need to process high levels of RS to survive. ATR and CHK1 inhibition can increase RS, leading to mitotic catastrophes that trigger cell death (98). Furthermore, inhibition of ATR-related signaling pathways can increase cell apoptosis and effectively improve tumor radiosensitivity (99). ATR inhibitors, such as AZD6738 and VE-821 as well as the CHK1 inhibitor SAR-020106 were effective radiosensitizers in preclinical studies (100–102). An ongoing phase I clinical trial (NCT03188965) is assessing the safety profile of ATR inhibitors (BAY1895344) (103).

#### RAD51

RAD51 is a master regulator of DNA replication and plays important roles in DSB repair, RS, and mitosis (104). RAD51 is a core factor in overcoming RS by slowing or stalling replication forks, which threatens replication integrity (105). It facilitates fork inversion, protects reverse forks, repairs and restarts broken replication forks, and post-replication gap filling (104, 106).

RAD51 inhibition may lead to increased tumor radiosensitivity, and it has been reported as a potential target of berberine in osteosarcoma radiosensitization (107). Valproate was found to increase tumor tissue cell radiosensitivity by increasing levels of RFWD3 and inhibiting RAD51 (108). The inhibition of nucleophosmin1 (NPM1) by YTR107, a small molecule that binds with NPM1, inhibits pentamer formation and represses RAD51 formation after IR. The synergistic effect of YTR107 and the PARP1/2 inhibitor ABT-888 increased RS and radiation-induced cell mortality (109).

#### BLM

BLM is a 3’-5’ ATP-dependent RecQ DNA helicase that is one of the most essential genome stabilizers involved in the regulation of DNA replication, recombination, and both homologous and non-homologous pathways of DSB repair (110). It interacts with topoisomerase IIIα (TOP3A), RecQ-mediated genome instability (RMI) 1, and RMI2 to form the BLM-Topoisomerase IIIα-RMI1-RMI2 (BTR) complex, which dissolves double Holliday junctions to produce non-crossover HR products. It also promotes DNA-end resection, restart of stalled replication forks, and processing of ultra-fine DNA bridges in mitosis (111). BLM helicase**-**deficient cells exhibit multiple defects in DNA replication, including accumulation of abnormal DNA replication intermediates, slower replication fork velocity, and excessive firing of dormant origins, thus exhibit increased levels of chromatid breakage and HR (112). It interacts directly with both RAD51 and RPA, and the function in DNA replication is regulated by sumoylation (113).

The high expression of BLM is a poor prognostic biomarker for multiple cancers (114, 115). Biallelic pathogenic variants in BLM cause bloom syndrome with severe pre- and postnatal growth deficiency, immune abnormalities, sensitivity to sunlight, insulin resistance, and a high risk for many cancers that occur at an early age (116). The symptoms of bloom syndrome including sensitivity to ultraviolet damage, which is similar to radiation, provide the possibility of transforming this genomic defect into a treatment sensitizer. ML216 is a small molecule inhibitor of BLM, and inhibits cell proliferation of BLM-proficient cells and increases the frequency of sister chromatid exchanges (117). Though there has been no data published on the links between a BLM inhibitor and radiation sensitivity till now, it is a promising target worth further research.

#### WEE1

When ssDNA accumulation at stalled replication forks activates ATR, it phosphorylates CHK1, which in turn activates WEE1 kinase and inhibits CDC25 phosphatase. While WEE1 inhibits CDKs, the key drivers of cell cycle progression, by phosphorylating the conserved threonine 14 (Thr14) and tyrosine 15 (Tyr15) residues, CDC25 activates CDKs by dephosphorylating the same residues (118). Elevated WEE1 expression reduces RS and activates G2/M checkpoints, conferring cell resistance to CHK1 inhibitors (98). Recently, it has been reported that the ATR-WEE1 module inhibits the MOS4-associated complex (MAC) to regulate RS responses (118).

The evidence suggests that WEE1 inhibition impairs the RS response activated by ATR, and thus increases tumor cell radiosensitivity (119). WEE1 kinase inhibitors sensitize tumor cells to proton and X-ray irradiation by inducing RS, independent of TP53 mutation status, such as AZD1775 (120–122). Clinical trials have shown that the WEE1 inhibitor adavosertib could potentiate the efficacy of RT; however, its clinical application is limited by its unfavorable safety profile (123).

### Targeting RS-Induced DDR

The RS response shares many biological pathways with DDR. They are widely intertwined and thus hard to completely distinguish (124). Here, we grouped the proteins that are typically related to the DDR pathway but are not necessarily involved in the RS response. Targeting these proteins usually impairs the DDR processing to enhance radiosensitivity, which makes them the most promising targets.

#### p53

The p53 signaling pathway plays a key role in determining radiosensitivity in normal tissues but is often inactivated during cancer. Loss of p53 in tumor cells allows them to escape cell cycle arrest and apoptosis checkpoints and promotes the growth of early-stage cancer cells by skipping the cell cycle checkpoint caused by RS (125). During DNA replication, IR-induced DNA damage stalls replication forks, and single-strand breaks (SSBs) can be transformed into DSBs, thereby activating the ataxia telangiectasia mutated (ATM)/ATR pathway. ATM and ATR phosphorylate p53 to increase its stability and activate target genes. RS induced by chemotherapy drugs such as trifluridine leads to cell senescence or apoptosis of tumor cells according to the state of p53 (126). Acetylation of p53 may modulate cancer cell radiosensitivity, which provides a promising strategy for radiosensitization (127).

#### MRE11

Meiotic recombination 11 (MRE11), the core of the MRE11/RAD50/NBS1 (MRN) complex, is involved in DNA break end detection, phosphorylation-dependent signal amplification, and DSB repair (128). The complex is critical for ATM activation of DSBs and downstream activation of G2/M and p53-dependent G1/S cell cycle checkpoints (129, 130). MRE11 also has endonuclease and exonuclease activities residing in the phosphodiesterase domain. These nuclease activities are crucial for the pathway choice of HR and NHEJ (131). Cancer cells rely on DNA repair for survival during cancer therapies, and thus MRE11 might be a promising synergistic therapeutic target.

Dysfunction of it in neoplastic breast tumors results in the accumulation of R-loops, replication-associated DSB, abundance of genomic deletions, and uncontrolled proliferation (132). Evidence suggests that its expression in cancer cells is critical for radioresistance (133). Low MRE11 expression in colorectal cancer cells reduced phosphorylated DNA-PKcs expression and further increases tumor radiosensitivity (134). There are different small and large molecular inhibitors targeting MRE11. Mirin is the first inhibitor found to specifically target MRE11 exonuclease activity with radiosensitizing properties (135). Lung cancer cells treated with Selenium, which is an essential trace element, showed decreased expression of MRE11 and significantly reduced colony formation relative to IR (136). OBP-301, with the insertion of the human telomerase reverse transcriptase (hTERT) promotor, also showed reduced MRE11 expression and thus enhanced radiosensitivity of lung cancer cells (137). Therefore, MRE11 inhibitors are clinically significant for enhancing radiosensitivity, and several clinical trials investigating their potential are ongoing (131).

#### ATM-CHK2

ATM kinase is a member of the PI3K-like protein kinase (PIKK) family with extensive roles in DDR signaling (138). Upon recruitment by the MRN complex to DSBs, ATM autophosphorylates at different serine sites resulting in the activation of CHK2, p53, and H2AX, which are involved in DNA repair processes and cell cycle arrest (139). The most important transducer of ATM signaling is CHK2, a kinase that signals to DNA repair, cell cycle arrest, and apoptosis. ATM phosphorylates CHK2 on threonine 68 (Thr68), thereby causing CHK2 dimerization and autophosphorylation of the kinase domain and is required for full activation (140).

ATM orchestrates the cellular DDR to cytotoxic DNA DSBs induced by radiation (141, 142). Overexpression of ATM indicates radiation resistance in breast cancer cells (143), whereas deficiency of ATM showed radiation sensitizer effects in multiple cancer types (144–147). Interestingly, more studies have focused on the radiation sensitizer effects that are dependent on the cell cycle and proliferation status (148, 149). After inhibition of proliferation, ATM status did not alter cell death or micronucleus formation after radiation, which suggests that ATM in endothelial cells was immaterial if a cell cycle block was present at the time of irradiation. It is consistent with other data showing that the effect of ATM on radiation sensitivity is more dependent on cell cycle regulation rather than the DDR pathway (148,149). Considering that ATM is a large protein with extensive regions of unknown function, the inhibition of its kinase activity may produce better synergistic effect on treatment. AZD0156, as a potent and selective bioavailable inhibitor of ATM, showed strong radiosensitizer effects *in vitro* and in a lung xenograft model (150). Specially, the ATM inhibitor AZD1390 is optimized for penetration of the blood-brain barrier with radiosensitizing effects on glioma and lung cancer cell lines, even in a brain metastasis model (141, 151). All of the evidence suggests that treatments targeting ATM may be promising in clinical trials.

#### MDM2

Mouse double minute 2 (MDM2) protein is a major negative regulator of p53 (152). When activated, p53 suppresses tumors in response to cell damage by mediating cell proliferation, cell cycle arrest, DNA repair, metabolism, angiogenesis, senescence, and apoptosis (153). In normal cells, the self-regulating feedback loop between MDM2 and p53 controls p53 expression (154, 155). The rescue of p53 function in cancer cells by inhibiting the interaction between p53 and MDM2 restored cycle arrest and apoptosis (156). Furthermore, inhibition of MDM2 phosphorylation leads to cell apoptosis and cell cycle arrest, thus repressing tumor cell proliferation in esophageal cancer cells (157). Additionally, MDM2 inhibitors, such as MI-219, increase tumor cell radiosensitivity in a p53-dependent manner. MI-219 combined with radiation resulted in increased p53-dependent DNA damage (158). A novel small-molecule inhibitor, APG-115, was found to enhance gastric adenocarcinoma cell radiosensitivity by blocking the interaction between MDM2 and p53 (159). Therefore, blocking the MDM2/p53 pathway has broad application prospects for treating tumors and enhancing tumor radiosensitivity, especially for tumors with low TP53 mutation levels, such as those of myeloid leukemia.

#### POLQ

DNA polymerase theta (POLQ) is a DNA polymerase that protects against error-prone transduction DNA synthesis and error-prone DSB (160). It is involved in a major DNA repair pathway that was initially named as alternative end-joining or microhomology-mediated end joining, and was later termed polymerase theta-mediated end joining because POLQ is indispensable in this process (161). POLQ overexpression reduces replication fork speed and impairs cell cycle progression (162). Furthermore, breast cancer related protein (BRCA) 2 and POLQ co-inhibition significantly improves tumor cell sensitivity to cisplatin (163). Reduced POLQ expression inhibits DSB repair and tumor cell survival. Several hepatocellular carcinoma cell lines (Huh7, HepG2, MHCC-92L, SK-HEP-1, and BEL-7404) with low POLQ expression after knockdown were found to be significantly sensitive to chemotherapeutic drugs (160). Depletion of POLQ in POLQ-dependent cancers (i.e., malignancies deficient in HR) leads to synthetic lethality. Furthermore, POLQ depletion was shown to synergize with PARP inhibition (164, 165), and the antibiotic novobiocin was recently reported as a selective POLQ inhibitor (166). Thus, combining novobiocin with radiotherapy should be a new research direction for targeting radioresistance.

#### BRCA

BRCAs (including BRCA1 and BRCA2) are thought to be the predominant proteins involved in HR in the DDR pathway. In addition, as a master regulator of HR, BRCA1 and BRCA2 also mediate fork protection (167, 168). BRCA mutations increase the susceptibility to various cancer types, including breast, ovarian, prostate, and pancreatic cancers (167). It is also well known that mutations in BRCA result in synthetic lethality with PARP inhibition. The underlying mechanism includes HR deficiency and increasing replication gaps. PARP inhibition results in replication fork collapse, chromosomal instability, cell cycle arrest in G2, and subsequent apoptosis in BRCA-deficient cells (169). Therefore, targeting PARP has become a reliable therapeutic strategy for eliminating BRCA1/2-mutated malignancies at diverse sites including the breast, primary peritoneum, fallopian tubes, ovaries, and pancreas (also see section 4.1.2) (170).

It has been reported that BRCA-deficient tumors are more sensitive to chemotherapeutic agents that induce RS (171). Furthermore, mutations in BRCA1/2 enhance radiosensitivity, indicating the possibility of BRCA as a biomarker of radiation sensitivity (172, 173). Since BRCA1/2 are both large proteins and have complex multiple functions, the development of inhibitors directly targeting BRCA1/2 is difficult to achieve. Therefore, PARPi has been suggested to patients with BRCA1/2 mutations for the synergistic lethal effects. The function of PARPi in radiosensitization are summarized in 4.2.1. Further research may focus on inhibitors that specifically affect the function of BRCA.

#### PI3K/AKT/mTOR

The PI3K/protein kinase B (AKT)/mammalian target of rapamycin (mTOR) pathway activates the downstream mediator mTOR to translate specific mRNA transcripts (174, 175). They synergistically work with CHK1 to repress DSB-induced RAD51 foci, thus impairing the HR process and enhancing RS in tumor cells. In addition, PI3K/mTORi slows the fork speed by increasing cell division cycle 45 homologue (CDC45) to promote a new origin of replication, thus enhancing CHK1-induced RS (176). The PI3K/AKT/mTOR signaling pathway is hyperactivated or altered in many cancer types (177). Inhibition of the pathway reduces tumor cell radioresistance (178, 179). For example, dactolisib, a dual PI3K/mTOR inhibitor, causes cell cycle arrest in the G2/M phase and improves the radiosensitivity of DU145 cell lines. Dactolisib also inhibits radiation-induced DSB repair in glioblastoma (GBM) cell lines by inhibiting DNA-PKcs and ATM and improves the radiosensitivity of radioresistant prostate cancer cell lines (180).

Torin 2 is a special class of PI3K pathway drugs, which not only inhibits the cell cycle at G1/S but also interferes with S phase progression, causing ssDNA accumulation, DNA damage, and increased checkpoint signaling in triple-negative BRCA cells (181). Furthermore, the dual PI3K/mTOR inhibitor apitolisib (GDC-0980) was demonstrated to inhibit growth and induce apoptosis in human GBM cells (182).

### Others

Despite all the classic proteins we discussed above, several novel concepts have been suggested in recent research. A large number of accessory factors involved in the assembly of replisomes have been reported, which includes multi-protein complexes that monitor replication fork progression, generate checkpoint and damage signals, and coordinate DNA synthesis with chromatin assembly (183). We list below several newly identified processes that may be related to RS and radiation sensitivity that may provide ideas for translating basic research into clinical trials.

#### Ubiquitin and SUMO

Post-translational modification of the DNA replication machinery by ubiquitin and small ubiquitin-like modifier (SUMO) plays key roles in cell division, DNA replication/repair, signal transduction, and cellular metabolism (184). Recent research revealed that ubiquitin/SUMO pathways are essential regulators of DNA replication during initiation, the S phase or elongation, and DNA replication termination (185). SUMO/ubiquitin equilibrium at active DNA replication forks controls CDK1 activation. An increase in ubiquitination of the replisome results in premature disassembly of the replication machinery and generation of CDK1-dependent DNA damage in the S phase (186).

Our group has identified ubiquitination factors that affect radiation sensitivity. We showed that ubiquitin-specific protease 9X (USP9X) mediates lysine-specific demethylase 4C (KDM4C) deubiquitination, which activates transforming growth factor-β2 (TGF-β2)/Smad/ATM signaling to promote radioresistance in lung cancer (142). Furthermore, ubiquitin-conjugating enzyme E2O (UBE2O) facilitates tumorigenesis and radioresistance by promoting MAX interactor 1 (Mxi1) ubiquitination and degradation (187). The SUMO-specific protease (SENP) pathway is also involved in tumor radiation sensitization (188). SUMO E3 ligase PIAS4, which is an essential signal for p53-binding protein 1 (53BP1) loading to the damage site, promote radiation resistance by increasing DDR (189). Ring finger protein 4 (Rnf4), an E3 ubiquitin ligase that targets SUMO-modified proteins, target SUMOylated mediators of DNA damage checkpoint protein 1 (MDC1) and SUMOylated BRCA1 loading at sites of DNA damage. Rnf4-deficient cells and mice exhibit increased sensitivity to IR by suppressing DDR (190). These findings identify ubiquitylation/SUMO as possible radiosensitization targets, but further research is needed.

#### UPR

UPR is the master regulator of endoplasmic reticulum (ER) stress. A deficiency in UPR results in apoptosis (191). Recent research revealed the link between hypoxia-induced RS and UPR (192). The induction of RNA/DNA helicase senataxin (SETX) in hypoxia is reliant on the protein kinase R (PKR)-like ER kinase (PERK)/activating transcription factor 4 (ATF4) arm of the UPR (32). Hypoxia is present in the majority of human tumors and is associated with poor prognosis due to the protection it affords to radiotherapy and chemotherapy (27). As we described earlier (section 3.1), anti-hypoxia treatments provide additional radiation benefits through cell apoptosis, which establishes a link between UPR and radiation sensitivity.

UPR is widely involved in the establishment and progression of cancers, including BRCA, prostate cancer, and GBM multiforme (193). Elevated mitochondrial UPR markers (mtHSP70 and HSP60) are associated with poor prognosis in patients with lung adenocarcinoma, which is activated by Maf1 through ATF5. Suppressing IR-induced mitochondrial UPR activation by rapamycin resulted in increased sensitivity to IR-mediated cytotoxicity (194). ONC201, an UPR activator, reduced oxidative phosphorylation and thus impairs cell cycle arrest, and the inhibition of DNA repair factors after radiation also enhanced radiation-induced cell death (34). As a new concept of radiosensitization, the clinical significance of UPR still requires further studies.

## RS-Induced Innate Immune Response in Radiation Sensitivity

Nowadays, the immune microenvironment is the hotspot in cancer research. It involves all processes of tumorigenesis, cancer progression, and treatment resistance. Innate immunity refers to nonspecific defense mechanisms that act immediately after antigen appearance. The activation of innate immune responses relies on pattern recognition receptors (PRRs). These PRRs detect endogenous damage-associated molecular patterns (DAMPs) or exogenous conserved pathogen-associated molecular patterns (PAMPs) to initiate a signaling cascade resulting in the production of interferons (IFNs) and inflammatory mediators (195, 196).

### RS-Induced Innate Immune Activation

As research progressed, some evidence revealed the relationship between RS and innate immune response, which plays a key role in cancer treatment resistance (197). In this study, we have summarized and discussed the potential relationship between targeting RS and innate immune activation.

#### Innate Immunity Activation by RS in Immune Cells

Excessive RS or RS deficiency leads to the accumulation of replication blockage-derived DNA in the cytoplasm or the formation of micronuclei, resulting in activating the cyclic GMP-AMP (cGAMP) and the cGAMP receptor stimulator of the interferon gene (STING) pathway. cGAMP synthase (cGAS) is a DNA sensor that recognizes and binds with DNA fragments in the cytoplasm, enabling cGAMP synthesis. cGAMP subsequently activates STING. The activation of STING further increases interferon regulatory factor 3 (IRF3) and nuclear factor kappa-light-chain-enhancer of activated B cell (NF-κB) levels (198). IRF3 and NF-κB act as transcription factors to trigger the transcription of IFN-I and cytokines (199). Apart from cGAS, γ-interferon-inducible protein-16, a cytosolic DNA sensor, can detect both self and non-self dsDNA to promote IRF3 and NF-κB-dependent interferon production *via* STING (195, 200, 201). IFN-1 plays a crucial role in both basal and therapeutic-induced immune responses to cancer. It is a potent immune cell activator, resulting in the activation and maturation of antigen presenting cells (198). The promotion of dendritic cell migration to the tumor site and their maturation depends on IFN-1 signaling (202, 203). Innate immune cells respond to IFN-1 by increasing antigen presentation and the production of immune response mediators, such as cytokines and chemokines. These events help in antigen presentation and chemokine production in innate cells as well as induce antibody production and enhance T-cell responses (198).

#### Innate Immunity Activation by RS in Tumor Cells

Innate immunity activation by tumor cells is a complex phenomenon. As we mentioned above, cancer cells usually experience higher RS, leading to more cytoplasmic DNA and micronuclei formation. They activate innate immunity by secreting IFN-1 *via* the cGAS-STING pathway, exocrine exosomes, or extracellular vesicles (EVs), which can be captured by immune cells for inducing a further immune response.

Cancer cells exposed to RS-inducing agents or deficient in RS response show the increased production of IFN-1 and proinflammatory cytokines that can foster an innate immune response (204, 205). One study found that the inhibition of the ATM/CHK2 DNA damage checkpoint axis led to excessive RS and cytosolic DNA accumulation, which subsequently activated the DNA sensor STING-mediated innate immune response in ARID1A-deficient tumors (206). Cytosolic DNA can also be released in exosomes or EVs (207, 208). Exosomes/EVs containing DNA works as DAMPs to innate immune cells. Study found that EVs and exosome dsDNA promoted inflammation *via* activating the STING pathway in macrophages (209).

The activation of STING in dendritic cells is essential for radiation-induced antitumor immunity (210). In contrast, cGAS-STING activation in tumor cells impairs HR in DDR, which promotes tumorigenesis (211). Moreover, cGAS can act as a decelerator of DNA replication forks, suppressing replication-associated DNA damage (212). The complex network mechanism made it hard to simply target or enhance cGAS-STING to reverse cancer treatment resistance. In contrast, high RS or RS-response deficiency always leads to simultaneous cell damage and immune activation. Hence, it would be a better choice for cancer treatment sensitization.

### Targeting RS Response Enhances Radiation Sensitivity by Innate Immunity

**Figure 5:**
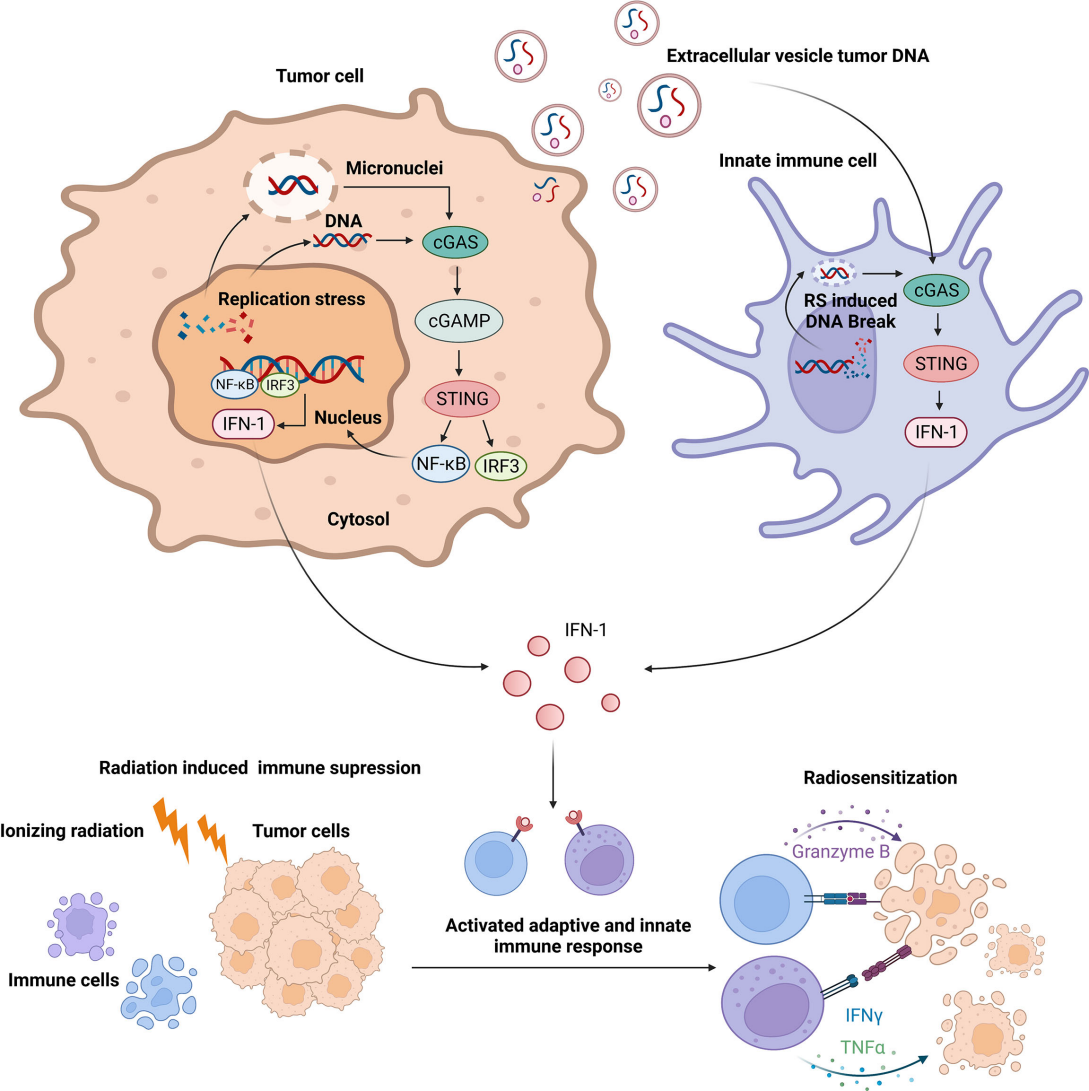
Replication stress-induced activation of innate immune response enhances radiosensitivity *via* cyclic GMP-AMP synthase–STING signaling.

The immune response caused by RT remains controversial. The inflammatory responses caused by RT are different depending on the RT pattern (213). Immune cells are highly radiosensitive compared with tumor cells (214). Conventional RT-induced myeloid-derived suppressor cell filtering leads to the suppressive tumor microenvironment (TME) rather than the active TME (215). Though the hypothesis that the damage signal released from tumor cells alone can activate a systemic antitumor immune response called the abscopal effect has been observed in a small-sample study (203), confirming the hypothesis without combining the signal with checkpoint inhibitors is difficult. The basic research revealed that RT may increase programmed death ligand 1 (PD-L1) levels in tumor and immune cells, contributing to immunosuppression and in part explaining the clinical success of the combination of RT with programmed cell death protein 1 (PD-1)/PD-L1 immunotherapy (216). As basic research data are available, more clinical trials regarding the combination of anti-PD-1/PD-L1 antibody with radiation are going on (217–220). Polymorphonuclear neutrophils recruited in the TME post-RT can facilitate tumor progression by forming neutrophil extracellular traps (221). Taken together, the tumor immune microenvironment is thought to be suppressed rather than activated after radiation, which plays a key role in radioresistance. Promoting immune response activation of TME is the key to enhance radiosensitivity (**Figure 5**).

Enhancing RS and targeting RS response are good choices to manage tumors. As mentioned above, excessive RS or RS response deficiency results in more DNA damage, which is the synergy effect of RS and RT from the direct tumor side. As they lead to dsDNA accumulation in the cytoplasm and cell apoptosis, which activate innate immunity, they may enhance radiation sensitivity from the indirect immune side (215, 222).

RAD51-depleted cells accumulate more cytosolic DNA after radiation, activating the STING pathway to increase innate immune response (223). ATR inhibition and radiation drive immune cell infiltration *via* tumor cell-intrinsic cytokine release to boost immunogenic response to radiotherapy and modulate the radiation-induced inflammatory TME (224). PARP inhibitor and radiation work synergistically to kill lung cancer cells by activating antitumor immunity in the form of increased CD8+ T lymphocytes and the activated STING/TANK-binding kinase 1/IRF3 pathway (225). WEE1 inhibitor increases tumor-specific cytotoxicity and shows a positive effect on immune response after radiation by dendritic cell activation, which can be combined with immune therapy (226, 227).

These studies indicate that the RS-induced activation of innate immune response may be crucial to enhance the radiosensitivity of tumor cells. However, more evidence is needed to draw a general conclusion. Moreover, further studies are needed on the interaction between the effect of RS-induced innate immune response on tumor-cell radiosensitivity and radiation-induced antitumor immunity to achieve the optimal radiotherapy efficacy.

## Conclusion

We have summarized the mechanisms of how RS response affects tumor radiosensitivity from the direct tumor side and indirect innate immune side and have further discussed potential targets and drugs to increase radiosensitization. We have reviewed several strategies including directly increasing RS, targeting RS response or RS-induced DDR, and other novel pathways. Although these strategies are predominantly based on preclinical evidence, they provide promising new ideas for enhancing radiosensitivity. As the relationship between RS and tumor radiosensitivity will be explored in the future, we expect these new strategies to bring substantial benefits to patients suffering from radioresistant malignancies.

## Data Availability Statement

The original contributions presented in the study are included in the article/supplementary material. Further inquiries can be directed to the corresponding author.

## Funding

This work was supported by the National Natural Science Foundation of China (No. 81802287 to RZ), College Student Innovation and Entrepreneurship Training Program (No. DYLC2021076 and S202110487427 to LW).

## Conflict of Interest

The authors declare that the research was conducted in the absence of any commercial or financial relationships that could be construed as a potential conflict of interest.

## Publisher’s Note

All claims expressed in this article are solely those of the authors and do not necessarily represent those of their affiliated organizations, or those of the publisher, the editors and the reviewers. Any product that may be evaluated in this article, or claim that may be made by its manufacturer, is not guaranteed or endorsed by the publisher.

## Abbreviations

RT, radiation therapy; RS, replication stress; DDR, DNA damage response; ATR, ataxia telangiectasia and rad3-related; CHK1, checkpoint kinase 1; ssDNA, single-stranded DNA; RPA, replication protein A; DSBs, double-strand breaks; UPR, unfolded protein response; HR, homologous recombination; T-LAK, T-lymphoid-activated killer; TOPK, T-LAK cell-derived protein kinase; Mcl-1, myeloid cell leukemia sequence 1; PARPs, poly (ADP-ribose) polymerases; IR, ionizing radiation; ATM, ataxia telangiectasia mutated; MRE11, meiotic recombination 11; MDM2, mouse double minute 2; POLQ, DNA polymerase theta; BRCA, breast cancer related protein; mTOR, mammalian target of the rapamycin; SUMO, small ubiquitin-like modifier; HIF, hypoxia inducible factor; RSF-1, spacing factor-1; NHEJ, non-homologous end joining; CDC, cell division cycle; SAC, spindle assembly checkpoint; APC/C, anaphase-promoting complex or cyclosome; PI3K, phosphoinositide 3-kinase; Bcl-2, B-cell lymphoma-2; Bax, Bcl-2-associated X protein; TAME, tosyl-L-arginine methyl ester; TOP3A, topoisomerase IIIα; RMI, RecQ-mediated genome instability; BTR, BLM-Topoisomerase IIIα-RMI1-RMI2; BLM, bloom syndrome helicase; MAC, MOS4-associated complex; MRN, MRE11/RAD50/NBS1; hTERT, human telomerase reverse transcriptase; RFWD3, RING finger and WD repeat domain 3; CHK1, checkpoint kinase 1; AKT, protein kinase B; mTOR, mammalian target of rapamycin; GBM, glioblastoma; USP9X, ubiquitin-specific protease 9X; KDM4C, lysine-specific demethylase 4C; TGF-β2, transforming growth factor-β2; UBE2O, ubiquitin-conjugating enzyme E2O; Mxi1, MAX interactor 1; SENP, SUMO-specific protease; 53BP1, p53-binding protein 1; Rnf4, Ring finger protein 4; MDC1, mediators of DNA damage checkpoint protein 1; ER, endoplasmic reticulum; SETX, senataxin; PKR, protein kinase R; PERK, PKR-like ER kinase; ATF4, activating transcription factor 4; PRRs, pattern recognition receptors; DAMPs, damage-associated molecular patterns; PAMPs, pathogen-associated molecular patterns; IFNs, interferons; cGAMP, cyclic GMP-AMP; STING, stimulator of the interferon gene; cGAS, cGAMP synthase; IRF3, interferon regulatory factor 3; NF-κB, nuclear factor kappa-light-chain-enhancer of activated B cell; EVs, extracellular vesicles; TME, tumor microenvironment; PD-L1, programmed death ligand 1; PD-1, programmed cell death protein 1.
